# Impact of 3D Radiotherapy With Cisplatin on the Eastern Cooperative Oncology Group Performance Status and Quality of Life in Terms of Acute Dermatological and Other Side Effects

**DOI:** 10.7759/cureus.89549

**Published:** 2025-08-07

**Authors:** Aliza Hamadani, Talha Laique, Ayesha Ahmad, Aina Mahmood, Rabia Mukhtar, Shazia Asim

**Affiliations:** 1 Dermatology, Ghurki Trust Teaching Hospital, Lahore, PAK; 2 Pharmacology, Sahara Medical College, Narowal, PAK; 3 Radiation Oncology, Ghurki Trust Teaching Hospital, Lahore, PAK; 4 Internal Medicine, Fatima Jinnah Medical University, Lahore, PAK; 5 Dermatology, Amna Inayat Medical Complex, Lahore, PAK; 6 Pharmacology, Lahore Medical and Dental College, Lahore, PAK

**Keywords:** acute side effects, cisplatin and 3d-concurrent radiotherapy, ecog status, head and neck cancer, quality of life

## Abstract

Introduction: The combination of 3D radiation therapy (3D-RT) with cisplatin has been the conventional treatment for squamous cell carcinoma of the head and neck for decades.

Aims: To assess the impact of 3D radiotherapy with cisplatin on the Eastern Cooperative Oncology Group (ECOG) performance status and quality of life in terms of acute side effects among head and neck cancer patients.

Methodology: After obtaining the hospital’s ethical approval, 106 head and neck cancer patients were enrolled at the Institute of Nuclear Medicine and Oncology, Lahore, Pakistan. Patients of both genders who were receiving radiotherapy or chemo-radiotherapy with stage I-III head and neck cancer with ECOG statuses 1 and 2 were enrolled. All patients received 70 grays in 35 fractions, with 2 grays per fraction, after a standard treatment of 40 mg/m^2^ cisplatin once weekly for seven weeks. All patients were followed up for assessment after one month post treatment. Patients who were included were evaluated once a month after radiation therapy and at weekly intervals during treatment. SPSS version 23 software (IBM Corp., Armonk, NY) was used to analyze the data. Chi-square and Fisher's exact test were applied, while a p-value ≤ 0.05 was taken as statistically significant.

Results: All patients experienced acute side effects after radiation therapy with cisplatin up to seven weeks into their treatment, including dermatitis, mucositis, xerostomia, and dysphagia of varying grades. After a month of treatment, however, these grade-related modifications subsided for all patients. A total of 39 patients (36.2%) had acceptable ECOG status, while 67 patients (63.8%) had poor ECOG status. An insignificant difference was observed in the frequency of acute adverse effect grades between poor and good ECOG status patients at the 1st, 4th, and 7th week of treatment. At the 11th week of treatment, the frequency of mucositis, dysphagia, and dermatitis grades between poor and good ECOG status patients showed a significant difference. The majority of the patients (n = 57, 53.8%) had a poor quality of life, while 27 (25.5%) had a good quality of life as they improved clinically.

Conclusion: Concurrent 3D-conformal radiotherapy with cisplatin in head and neck cancer patients is associated with significant acute dermatological and mucosal toxicities, leading to temporary deterioration in the ECOG performance status and quality of life. However, most patients showed recovery in functional status post treatment.

## Introduction

Squamous cell cancer of the head and neck is a diverse illness that can involve any part of the oral cavity, such as the larynx, lip, oral cavity, nasopharynx, oropharynx, hypopharynx, and oropharynx [1]. With 630,000 new cases recorded each year, squamous cell carcinoma of the head and neck region ranks sixth among all malignancies worldwide. The five-year survival rate for people with head and neck cancer is between 45% and 55% [2]. In Pakistan, the prevalence of head and neck malignancies is higher among men (21%) than among women (11%) [3,4].

According to a prior report, head and neck malignancies have a significant mortality rate, accounting for about 300,000 fatalities annually [5]. Early-stage illness patients typically exhibit nebulous signs and symptoms. They are contingent upon the principal site of illness [6]. If the oral cavity is affected, patients experience painful, non-healing ulcers; if the oropharynx is involved, symptoms include otalgia, chronic dysphagia, and sore throat. On the other hand, when the disease progresses, patients typically have a neck mass with supraglottic tumors. There is a high correlation between head and neck cancer and other risk factors, including genetics, environmental variables, occupation, and selected lifestyle choices, such as drinking alcohol and smoking cigarettes [7]. This illness has surfaced more lately due to different strains of the human papillomavirus (HPV 16, 18) [8,9]. As a result of reduced tobacco usage, some nations have reported a decline in the incidence of cancer of the oral cavity [10]. Other treatment options for head and neck cancers include surgery, brachytherapy, proton beam therapy, photodynamic therapy, and immunotherapy, as reported by previous studies [7,9].

An adverse event (AE) is any unwanted effect produced as a result of a medical treatment or procedure. Cisplatin use was associated with increased toxicities in head and neck cancer patients. Toxicity criteria of the Radiation Therapy Oncology Group (RTOG) and the European Organization for Research and Treatment of Cancer (EORTC) classified acute side effects like mucositis, dysphagia, dermatitis, and dry mouth (xerostomia) into grades to show organ toxicity among subjects receiving radiation therapy (RT) [6].

The combination of 3D radiation therapy (3D-RT) with cisplatin has been the regimen of choice for the treatment of head and neck cancer for decades, as it kills tumor cells maximally, followed by the development of acute and chronic side effects during the treatment period. As there is an increasing number of head and neck cancer cases among our population, we planned the current project with the aim to assess the impact of 3D radiotherapy with cisplatin on the Eastern Cooperative Oncology Group (ECOG) performance status and quality of life (QoL) in terms of acute side effects like dermatitis, mucositis, dysphagia, and xerostomia among head and neck cancer patients. This study helped us in the proper assessment of ECOG status and QoL among patients who received 3D-RT with cisplatin. Secondly, this study helped us to design a protocol for the proper management of acute side effects to reduce the illness, as it provided useful insights about the disease.

## Materials and methods

This descriptive case series was conducted in the Department of Radiation Oncology at the Institute of Nuclear Medicine and Oncology (INMOL), Lahore, Pakistan. After obtaining the hospital’s ethical approval, 106 head and neck cancer patients were enrolled through non-probability, consecutive sampling at INMOL, Lahore, Pakistan. All patients received 70 grays in 35 fractions, with 2 grays per fraction, after a standard treatment of 40 mg/m² cisplatin once weekly for seven weeks. The enrolled patients were assessed weekly during treatment and once a month following radiation therapy. At the time of enrollment, every baseline metric was included in a performance evaluation. The acute side effects of the patients were closely monitored. Toxicity criteria of the RTOG and the EORTC classified acute side effects like mucositis, dysphagia, dermatitis, and dry mouth (xerostomia) into grades to show organ toxicity among subjects receiving RT. These toxicity criteria were followed in the present study. Written informed consent was taken from participants at the time of enrollment, and the whole methodology was explained to them. QoL was assessed through the EORTC Quality of Life Questionnaire - Head and Neck 35 (QLQ-H&N35). 

EORTC QLQ-H&N35

The questionnaire covered domains such as pain, swallowing, saliva, taste, speech, social eating, social contact, and sexuality. Each domain's scores ranged from 0 to 100. The higher the score, the worse the outcome. This questionnaire is commonly used in our setup (INMOL), as its validity has been documented previously [11].

All patients (both genders) with head and neck carcinoma who were receiving radiotherapy or chemo-radiotherapy with stage I-III head and neck cancer with ECOG statuses 1 and 2, aged 25-75 years, were enrolled. Head and neck carcinoma patients who did not complete their treatment, had pre-existing skin diseases on their medical record, or had any second malignancy, metastasis, or recurrent disease were excluded at the time of enrollment.

Statistical analysis

SPSS version 23 software (IBM Corp., Armonk, NY) was used to analyze the data. Descriptive statistics were applied to summarize study variables. Quantitative variables like age and total radiation dosage were provided by mean ± SD. Qualitative factors such as patient ECOG status, gender, dental hygiene, and QoL were represented by frequencies and percentages.

For interferential analysis, the chi-square test was used to evaluate the association between categorical variables. Fisher's exact test was applied to ensure statistical validity. A p-value of <0.05 was considered statistically significant, indicating a meaningful association between the studied variables.

## Results

Age among the enrolled participants was described by mean ± SD as 57.8 ± 8.3 years, while total radiation dose was 62.8 ± 7.4 grays. The most common site was the oral cavity (31, 29.2%), followed by the pharynx (23, 21.7%), hypopharynx (22, 20.8%), nasopharynx (22, 20.8%), and larynx (8, 7.5%), among all enrolled head and neck carcinoma patients. There were 52 (49.1%) patients with good oral hygiene, 28 (26.4%) patients with average oral hygiene, and poor hygiene was observed in 26 (24.5%) patients (Table 1). The majority of the patients (57, 53.8%) had a poor QoL, while 27 (25.5%) had a good QoL as they improved clinically. Fortunately, no drop out as well as no mortality was reported among participants enrolled in the current project.

**Table 1:** Qualitative variables of enrolled patients (n = 106).

Variable	Category	Frequency (n), percentage (%)
Sex	Male	44, 41.5%
	Female	62, 58.5%
Performance status	Bad	67, 63.8%
	Good	39, 36.2%
Oral hygiene	Bad	26, 24.5%
	Normal	28, 26.4%
	Good	52, 49.1%
Quality of life	Poor	57, 53.8%
	Good	27, 25.5%
	Average	22, 20.8%

All enrolled patients developed acute side effects of varying grades both during and post treatment (Table 2). During treatment with 3D-RT and cisplatin, all mentioned acute side effects worsen from grade 1 to grade 4. However, these side effects improved with a declining trend in their severity in the follow-up post-treatment period, with minimal improvement seen in xerostomia grades.

**Table 2:** Acute side effects of varying grades during pre and post treatment. Data are represented as frequency (n) and percentage (%).

Parameters	Week	Grade 0	Grade 1	Grade 2	Grade 3	Grade 4	Grade 5
Grades of dermatitis	1^st^	98 (92.5%)	8 (7.5%)	Nil	Nil	Nil	Nil
	4^th^	Nil	67 (63.2%)	31 (29.2%)	8 (7.5%)	Nil	Nil
	7^th^	0 (0%)	6 (5.7%)	8 (7.5%)	84 (79.2%)	8 (7.5%)	0 (0%)
	11^th^	0 (0%)	14 (13.2%)	53 (50%)	39 (36.8%)	0 (0%)	0 (0%)
Grades of mucositis	1st	79 (74.5%)	27 (25.5%)	0 (0%)	0 (0%)	0 (0%)	0 (0%)
	4^th^	0 (0%)	78 (73.6%)	28 (26.4)	0 (0%)	0 (0%)	0 (0%)
	7^th^	0 (0%)	0 (0%)	52 (49.1%)	49 (46.2%)	5 (4.7%)	0 (0%)
	11^th^	56 (52.8%)	49 (46.2%)	1 (0.9%)	0 (0%)	0 (0%)	0 (0%)
Grades of dysphagia	1^st^	106 (100%)	0 (0%)	0 (0%)	0 (0%)	0 (0%)	0 (0%)
	4^th^	0 (0%)	65 (61.3%)	41 (38.7%)	0 (0%)	0 (0%)	0 (0%)
	7^th^	0 (0%)	0 (0%)	19 (17.9%)	47 (44.3%)	40 (37.7%)	0 (0%)
	11^th^	42 (39.6%)	24 (22.6%)	40 (37.7%)	0 (0%)	0 (0%)	0 (0%)
Grades of xerostomia	1^st^	78 (73.6%)	28 (26.4%)	0 (0%)	0 (0%)	0 (0%)	0 (0%)
	4^th^	10 (9.4%)	68 (64.2%)	18 (17%)	10 (9.4%)	0 (0%)	0 (0%)
	7^th^	0 (0%)	0 (0%)	29 (27.4%)	20 (18.9%)	57 (53.8%)	0 (0%)
	11^th^	0 (0%)	0 (0%)	69 (65.1%)	37 (34.9%)	0 (0%)	0 (0%)

Chi-square and Fisher's exact tests were used to compare the frequency of mucositis grades between poor and good ECOG status. At the 11th week of treatment, the frequency of mucositis grades between poor and good ECOG status patients showed a significant difference (Table 3).

**Table 3:** Comparison of grades of mucositis between ECOG status. * Significant. The chi-square test was applied. Data are represented as frequency (n) and percentage (%). ECOG: Eastern Cooperative Oncology Group.

Week	ECOG	Grade 0	Grade 1	Grade 2	Grade 3	Grade 4	Grade 5	p-value
1^st^	Poor	47 (70.1%)	20 (29.9%)	0 (0%)	0 (0%)	0 (0%)	0 (0%)	0.175
	Good	32 (82.1%)	7 (17.9%)	0 (0%)	0 (0%)	0 (0%)	0 (0%)	
4^th^	Poor	0 (0%)	46 (68.7%)	21 (31.3%)	0 (0%)	0 (0%)	0 (0%)	0.131
	Good	0 (0%)	32 (82.1%)	7 (17.9%)	0 (0%)	0 (0%)	0 (0%)	
7^th^	Poor	0 (0%)	0 (0%)	29 (43.4%)	35 (52.2%)	3 (4.5%)	0 (0%)	0.279
	Good	0 (0%)	0 (0%)	23 (59.0%)	14 (35.9%)	2 (5.1%)	0 (0%)	
11^th^	Poor	0 (0%)	32 (47.8%)	35 (52.2%)	0 (0%)	0 (0%)	0 (0%)	0.001*
	Good	0 (0%)	24 (61.5%)	14 (35.9%)	1 (2.6%)	0 (0%)	0 (0%)	

Chi-square and Fisher's exact tests were used to compare the frequency of dysphagia grades between poor and good ECOG status. At the 11th week of treatment, a significant difference was observed in the frequency of dysphagia grades between the poor and good ECOG status patients (Table 4).

**Table 4:** Comparison of grades of dysphagia between ECOG status. * Significant. The chi-square test was applied. Data are represented as frequency (n) and percentage (%). ECOG: Eastern Cooperative Oncology Group.

Week	ECOG	Grade 0	Grade 1	Grade 2	Grade 3	Grade 4	Grade 5	p-value
1^st^	Poor	67 (100.0%)	0 (0%)	0 (0%)	0 (0%)	0 (0%)	0 (0%)	-
	Good	39 (100.0%)	0 (0%)	0 (0%)	0 (0%)	0 (0%)	0 (0%)	
4^th^	Poor	0 (0%)	38 (56.7%)	29 (43.3%)	0 (0%)	0 (0%)	0 (0%)	0.202
	Good	0 (0%)	27 (69.2%)	12 (30.8%)	0 (0%)	0 (0%)	0 (0%)	
7^th^	Poor	0 (0%)	0 (0%)	11 (16.4%)	27 (40.3%)	29 (43.3%)	0 (0%)	0.303
	Good	0 (0%)	0 (0%)	8 (20.5%)	20 (51.3%)	11 (28.2%)	0 (0%)	
11^th^	Poor	0 (0%)	20 (29.9%)	18 (26.9%)	29 (43.4%)	0 (0%)	0 (0%)	0.026*
	Good	0 (0%)	22 (56.4%)	6 (15.4%)	11 (28.2%)	0 (0%)	0 (0%)	

Chi-square and Fisher's exact tests were used to compare the frequency of dermatitis grades between poor and good ECOG status. At the 11th week of treatment, the frequency of dermatitis grades showed a significant difference between the poor and good ECOG status patients (Table 5).

**Table 5:** Comparison of grades of dermatitis between ECOG status. * Significant. The chi-square test was applied. Data are represented as frequency (n) and percentage (%). ECOG: Eastern Cooperative Oncology Group.

Week	ECOG	Grade 0	Grade 1	Grade 2	Grade 3	Grade 4	Grade 5	p-value
1^st^	Poor	63 (94.0%)	4 (6.0%)	0 (0%)	0 (0%)	0 (0%)	0 (0%)	0.462
	Good	35 (89.7%)	4 (10.3%)	0 (0%)	0 (0%)	0 (0%)	0 (0%)	
4^th^	Poor	0 (0%)	43 (64.2%)	20 (29.9%)	4 (6.0%)	0 (0%)	0 (0%)	0.731
	Good	0 (0%)	24 (61.5%)	11 (28.2%)	4 (10.3%)	0 (0%)	0 (0%)	
7^th^	Poor	0 (0%)	5 (7.5%)	3 (4.5%)	55 (82.1%)	4 (6.0%)	0 (0%)	0.257
	Good	0 (0%)	1 (2.6%)	5 (12.8%)	29 (74.4%)	4 (10.3%)	0 (0%)	
11^th^	Poor	0 (0%)	8 (11.9%)	35 (52.2%)	24 (35.8%)	0 (0%)	0 (0%)	0.058*
	Good	0 (0%)	6 (15.4%)	18 (46.2%)	15 (38.5%)	0 (0%)	0 (0%)	

Chi-square and Fisher's exact test were used to compare the frequency of xerostomia grades between poor and good ECOG status. At the 11th week of treatment, the frequency of xerostomia grades was also similar between the poor and good ECOG status patients (Table 6).

**Table 6:** Comparison of grades of xerostomia between ECOG status. The chi-square test was applied. Data are represented as frequency (n) and percentage (%). ECOG: Eastern Cooperative Oncology Group.

Week	ECOG	Grade 0	Grade 1	Grade 2	Grade 3	Grade 4	Grade 5	p-value
1^st^	Poor	49 (73.1%)	18 (26.9%)	0 (0%)	0 (0%)	0 (0%)	0 (0%)	0.890
	Good	29 (74.4%)	10 (25.6%)	0 (0%)	0 (0%)	0 (0%)	0 (0%)	
4^th^	Poor	7 (10.4%)	42 (62.7%)	11 (16.4%)	7 (10.4%)	0 (0%)	0 (0%)	0.918
	Good	3 (7.7%)	26 (66.7%)	7 (17.9%)	3 (7.7%)	0 (0%)	0 (0%)	
7^th^	Poor	0 (0%)	0 (0%)	19 (28.4%)	11 (16.4%)	37 (55.2%)	0 (0%)	0.699
	Good	0 (0%)	0 (0%)	10 (25.6%)	9 (23.1%)	20 (51.3%)	0 (0%)	
11^th^	Poor	0 (0%)	0 (0%)	44 (65.7%)	23 (34.3%)	0 (0%)	0 (0%)	0.870
	Good	0 (0%)	0 (0%)	25 (64.1%)	14 (35.9%)	0 (0%)	0 (0%)	

## Discussion

This study assessed the acute impact of concurrent 3D-conformal radiotherapy (3D-CRT) with cisplatin on ECOG performance status and QoL in patients with head and neck squamous cell carcinoma (HNSCC), with a specific focus on dermatological and mucosal toxicities in our clinical setting at INMOL, Lahore, Pakistan. Our findings are consistent with global literature, highlighting that although this treatment strategy is effective, it is associated with considerable acute toxicity that affects patient functionality and well-being.

Sadly, the high rate of head and neck cancer in our population has had a negative psychological and social impact on our society. However, because we are a developing nation with few human resources, this health problem has not been adequately addressed in our systems. False beliefs about medications administered at oncology centers and alternative treatment systems, such as traditional medicines, that divert patients from receiving treatment for the intended length of time are causes of therapeutic failure for both chemotherapy and radiation therapy [9]. Thus, the burden of disease in society is increased by this mindset. Additionally, individuals must deal with serious acute and chronic toxicity associated with their treatment.

In the current project, there was an increase in grades of dermatitis, mucositis, dysphagia, and xerostomia among patients with treatment given, as results depicted that at the 7th week of treatment, moderate dermatitis (grade 3) was observed in 79.2%, moderate mucositis (grade 3) was observed in 46.3%, severe dysphagia (grade 4) developed in 37.7%, and severe xerostomia (grade 4) was observed in 53.8% of patients, as shown in Table 2. However, there was a decline in the grades of acute side effects post treatment (11th week) among all patients, as shown in Table 2. Our findings showed that acute adverse effects like mucositis, dysphagia, xerostomia, dermatitis, nausea, and vomiting occur during treatment but improve post radiation (follow-up period) if appropriately and consistently monitored, as they were fully validated by earlier research on the use of chemo-radiotherapy for head and neck cancer [12]. According to earlier research, acute adverse effects decreased one month following radiation treatment [13]. We observed that nearly 63.8% of patients (n = 67) experienced a temporary deterioration in ECOG performance status during treatment, particularly between weeks four and six of therapy. There were 52 (49.1%) patients with good oral hygiene, 28 (26.4%) patients with average oral hygiene, and poor hygiene was observed in 26 (24.5%) patients (Table 1). This aligns with data from one previous study, which reported that 48% of patients undergoing concurrent chemoradiation showed a decline of at least one point in ECOG performance by week five, with gradual improvement by six and eight weeks post treatment [14]. The performance decline is attributable to the cumulative effect of mucositis, dysphagia, fatigue, and skin toxicity, all of which directly affect oral intake, hydration, and general activity levels. Our results support the necessity of supportive interventions such as early nutritional support and pain control, as recommended in the National Comprehensive Cancer Network (NCCN) guidelines for supportive care in head and neck cancer patients. Similarly, one previous study showed that the majority of their enrolled patients (57%) with localized head and neck cancer had poor ECOG status post treatment [8].

Similarly, the majority of the patients (53.8%) had a poor QoL, 25.5% had a good QoL as they improved clinically, but 20.8% had an average QoL (Table 1) in the present study. One researcher reported that the most affected domains during chemo-radiotherapy were pain, swallowing, and fatigue, with partial recovery by three months post treatment [14]. Our study also showed a rebound in QoL metrics within eight weeks post therapy, supporting the idea that these toxicities are largely acute and reversible. The poor QoL was supported by the development of acute side effects from 3D-RT with the cisplatin treatment regimen, as depicted by many previous studies on head and neck cancer treatment [12-16]. Cisplatin use has been associated with the development of acute side effects of varying grades.

Although QOL outcomes were measured using a validated tool, interpretation of these results was limited by the absence of a control/comparison group. Thus, without baseline data or comparison with an untreated or differently treated group, it is difficult to attribute observed QoL patterns solely to treatment intervention. However, patients tolerated therapy without permanent disability, which is crucial in curative-intent treatments.

Counseling patients about the expected side effect timeline may improve compliance and reduce psychological distress. However, new treatment modalities like transoral robotic surgery, proton beam therapy, targeted therapy, de-intensified radiation therapy, and immunotherapy affect QoL minimally among patients undergoing treatment for head and neck cancer. More research using these modalities is still needed for their validated results on a larger scale.

Patients with squamous head and neck cancer frequently receive radiochemotherapy using regimens based on cisplatin, rather than regimens that contain cisplatin only. Throughout the course of treatment, all patients underwent chemo-radiation with cisplatin at a dose of 40 mg/m²/week. The current investigation showed effective local and regional control and no deaths. Similar to this, a previous study examined two distinct cisplatin dosages, five days a week for four weeks (20 mg/m²/day for the lower dose and 25 mg/m²/day for the higher dose). They came to the conclusion that cancer patients responded better to smaller doses, which were well tolerated. Their results therefore confirmed our findings that a smaller dose of cisplatin is significantly more effective for a longer duration of treatment [10,17].

The findings of this study align with previous research indicating that chemoradiotherapy, particularly cisplatin-based regimens, is associated with significant acute toxicities and a decline in QoL domains, such as swallowing, speech, and social functioning. However, in the absence of a control group, these results must be interpreted with caution.

A recent study confirmed that concurrent chemoradiotherapy significantly affects QoL, especially in the early post-treatment phase, but noted partial recovery in some domains within six to 12 months [18]. Similarly, global population-based studies from high-income settings have shown that advancements like intensity-modulated radiotherapy (IMRT) are associated with better functional outcomes and lower xerostomia rates compared to 3D-RT.

Limitations

Our study had a number of limitations, including a single-center study, a small sample size, short-term follow-up, and a lack of genetic studies, followed by financial constraints. Long-term toxicities such as fibrosis, osteo-radio-necrosis, and permanent xerostomia were also not assessed. In addition, the study relied on self-reported QoL assessments without validation from patient caregivers. Interpretation of these results was limited by the absence of a control/comparison group; thus, findings should be considered descriptive rather than conclusive.

However, more studies with larger sample sizes using validated assessment tools are recommended, with long-term follow-ups of six months or one year to observe chronic and permanent side effects affecting QoL. More comparison studies are recommended using new treatment modalities to see their impact on clinical, psychological outcomes involving QoL.

## Conclusions

Concurrent 3D-CRT with cisplatin remains a widely used and effective treatment modality for patients with locally advanced head and neck cancers, particularly in resource-constrained settings. However, our study reinforces that this approach is associated with significant acute toxicities, particularly dermatological (radiation dermatitis) and mucosal (oral mucositis, dysphagia, xerostomia) complications. These adverse effects contribute to a temporary decline in ECOG performance status and a measurable deterioration in various domains of health-related QoL, especially during the peak treatment period.

Despite the severity of these acute effects, the majority of patients demonstrated gradual recovery of function and QoL post treatment, indicating the transient nature of these toxicities when appropriately managed. This emphasizes the resilience of patients and the critical role of early supportive interventions, including nutritional support, pain management, hydration, and skin care protocols. This study highlighted the impact of cancer treatment (3D-RT) on functional status and QoL of head and neck cancer patients, as these parameters usually remain untouched in our society.
